# Phase Transformation Behaviors of Medium Carbon Steels Produced by Twin Roll Casting and Compact Strip Production Processes

**DOI:** 10.3390/ma16051980

**Published:** 2023-02-28

**Authors:** Shaohua Li, Haibo Feng, Shuize Wang, Junheng Gao, Haitao Zhao, Honghui Wu, Shuai Xu, Qingxiao Feng, Hualong Li, Xinyuan Liu, Guilin Wu

**Affiliations:** 1 Innovation Research Institute for Carbon Neutrality, University of Science and Technology Beijing, Beijing 100083, China; 2 Institute of Research of Iron and Steel, Shasteel, Zhangjiagang 215625, China

**Keywords:** twin roll casting, compact strip production, segregation, decarburization, phase transformation

## Abstract

Medium carbon steels have been widely used in the fields of tool and die manufacturing due to their outstanding hardness and wear resistance. In this study, microstructures of 50# steel strips fabricated by twin roll casting (TRC) and compact strip production (CSP) processes were analyzed to investigate the influences of solidification cooling rate, rolling reduction, and coiling temperature on composition segregation, decarburization, and pearlitic phase transformation. The results show that a partial decarburization layer with a thickness of 13.3 μm and banded C-Mn segregation were observed in the 50# steel produced by CSP, leading to the banded distributions of ferrite and pearlite in the C-Mn poor regions and C-Mn rich regions, respectively. For the steel fabricated by TRC, owing to the sub-rapid solidification cooling rate and short processing time at high temperatures, neither apparent C-Mn segregation nor decarburization was observed. In addition, the steel strip fabricated by TRC has higher pearlite volume fractions, larger pearlite nodule sizes, smaller pearlite colony sizes and interlamellar spacings due to the co-influence of larger prior austenite grain size and lower coiling temperatures. The alleviated segregation, eliminated decarburization and large volume fraction of pearlite render TRC a promising process for medium carbon steel production.

## 1. Introduction

Medium carbon steels have high hardenability, superior hardness and good wear and contact fatigue resistance, which facilitate their wide application in the production of tools and dies [1,2,3]. However, to improve the quality of medium carbon steels, there are several technical issues requiring to be addressed. The addition of high contents of carbon and other alloying elements in these steels results in serious composition segregation and surface decarburization of the hot-rolled products. It has been reported that composition segregation and decarburization are detrimental to the impact toughness, fatigue life, wear resistance and other properties, which are critical for the service performances of medium carbon steels [4,5,6,7]. Therefore, addressing the composition segregation and surface decarburization issues is essential to enhance the service performance of medium carbon steels. Composition segregation significantly influences the type and distribution of microstructure in the hot-rolled sheet. For example, in contrast to a random distribution of proeutectoid ferrite and pearlite, C and Mn segregation lead to the alternating distributions of proeutectoid ferrite and pearlite [8,9,10,11]. Low solidification cooling rates will introduce severe composition segregation in slabs, which causes the formation of distinct segregation bands in the as-hot-rolled microstructure [12,13,14]. Although the solidification cooling rate of CSP process reaches 10~100 K/s [15], composition segregation is still frequently observed in the hot-rolled strips [8]. In addition, decarburization layers are observed as well, which reduce the surface hardness and thus deteriorate the wear and fatigue resistance of the steel [16,17]. The extent of surface decarburization of hot-rolled strips is subjected to the influences of the following factors, including alloying elements [18,19,20], atmosphere [3,21,22], reheating temperature and holding time [2,3,23,24,25,26,27] and so on. Due to the long soaking time at high temperatures during the production process of CSP and the subsequent multi-pass hot rolling at high temperatures, extensive surface decarburization usually occurs in the hot-rolled strips [28,29]. The TRC is a typical near-net-shape steel manufacturing technology, which is mainly used to produce ultra-thin hot-rolled strips. Owing to the characteristics of direct rolling and single-pass rolling of the TRC process, the high temperature exposure time of the strips can be significantly shortened, which can effectively reduce the extent of surface decarburization of medium/high carbon steels. In addition, the solidification cooling rate of the TRC process is in the range of 10^2^ ~ 10^3^ K/s, which is about 100–1000 times higher than that of the conventional continuous casting process and about 10–100 times higher than that of the CSP process [30]. It has been reported that sub-rapid solidification (such as the TRC process) can effectively solve the composition segregation issue of high strength low alloy steels and high Mn steels [31]. Furthermore, in comparison with the conventional casting and rolling process, the TRC process can reduce energy consumption by more than 85%, so the TRC process also has economic and environmental advantage, especially under the background of carbon neutralization [32,33].

At present, although great efforts have been devoted into expanding the production capability of the TRC process to cover more steel grades such as low-carbon steels, stainless steels, silicon steels, etc. [34], there is little researches regarding the production of medium or high carbon steels through the TRC process. As mentioned above, the typical characteristics of the TRC process are the sub-rapid solidification cooling rate, direct rolling and single-pass rolling, which can affect the composition segregation, surface decarburization, prior austenite grain size (PAGS) [35,36], and pearlitic phase transformation of the hot-rolled strips. More specifically, the rolling reduction has a great influence on the PAGS, which has a vital impact on the pearlite nodule size (PNS) [35,37,38,39,40] and pearlite colony size (PCS) [41,42,43,44,45]. Pearlite interlamellar spacing (IS) mainly depends on the coiling temperature (isothermal transformation temperature) [38,41,45,46,47,48]. Therefore, it is of significance to investigate the feasibility of the production of medium carbon steels using the TRC process.

In this paper, 50# steel samples were prepared by TRC and CSP processes, and the extent of composition segregation and surface decarburization of the two samples were systematically investigated and compared. In addition, the effects of composition segregation, rolling reduction and coiling temperature on the phase transformation behaviors of the two strips were investigated and discussed in detail.

## 2. Materials and Methods

**Figure 1:**
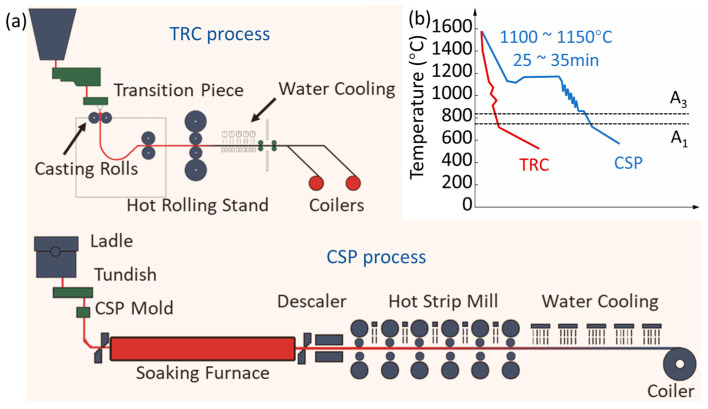
(**a**) Schematic diagrams showing the procedures of the TRC and CSP processes, respectively. (**b**) The thermo-mechanical schedules of the strips produced by the TRC and CSP processes. A_1_ is the eutectoid temperature, and A_3_ indicates the temperature at which single-phase austenite begins to transform into ferrite forming a mixture of austenite and ferrite during cooling.

The hot-rolled strips were taken from TRC and CSP processes, respectively. The schematic diagrams and thermo-mechanical schedules of the TRC and CSP processes are presented in Figure 1. Compared with the CSP process, the TRC process is characterized by a higher casting speed, higher cooling rate, thinner casting slab thickness and single-pass hot rolling. The primary technical parameters for the CSP and TRC processes are listed in Table 1. The chemical compositions of the three hot-rolled strips are shown in Table 2. The total rolling reduction and coiling temperature are listed in Table 3. The nomenclature of the samples of the TRC in Table 2 and Table 3 is determined by the rolling reduction. The characteristics of the as-hot-rolled strips, including the composition segregation, the decarburization layer thickness, the volume fraction of proeutectoid ferrite (VFPF), PAGS, PNS, PCS and IS, were analyzed quantitatively. Composition segregation of the CSP-50# and TRC-50#-10 was revealed by a mixed solution of 15 mL supersaturated picric acid solution and detergent. An optical microscope (OM) was used to characterize the composition segregation, surface decarburization and microstructure of the studied steel strips. Element distributions on the longitudinal section (the section containing the rolling direction, RD, and the normal direction, ND) of the two hot-rolled strips were analyzed by electron probe microanalysis (EPMA, EPMA-1720H). The thicknesses of the surface decarburization layers of the three strips were characterized by EPMA, and five C concentration profiles were measured from the surface to the interior of each sample. The supersaturated picric acid aqueous solution and 2% nital were both used to reveal the ferrite-pearlite microstructure. The volume fraction of proeutectoid ferrite was quantified by Image J. Proeutectoid ferrite can effectively outline the prior austenite grain boundary (PAGB), and PAGS was measured by the random intercept method. Scanning electron microscope (SEM, ZEISS Gemini SEM 500) was used to analyze the pearlite microstructure. The average PCS and average IS were evaluated. At least 500 measurements were taken for each sample. Electron backscattered diffraction (EBSD, EDAX OIM 6.0) equipped in a SEM (ZEISS Gemini SEM 500) was utilized to characterize the pearlite nodule. EBSD specimens were prepared by electro-polishing at −20 °C in a solution of perchloric acid and alcohol (1:9 in volume) with a voltage of 15 V for 15 s by following the standard metallographic preparation procedures. EBSD data-processing was also conducted using Oxford^®^ HKL Channel 5 system with a step size of 0.4 μm. Misorientation angles ≥ 15° were defined as high angle grain boundaries for determining the PNS. Transmission electron microscope (TEM, JEOL JEM 2100) was adopted to reveal the fine structure of pearlite. TEM samples were grounded to approximately 50 μm thick with 2000 grade silicon papers, followed by punching to 3 mm disks. TEM foils were prepared by twin-jet polishing at −30 °C in a solution identical to the electro-polishing solution for EBSD samples. The Scheil solidification model in Thermo-Calc software was used to calculate the composition segregation profiles across a dendrite of 50# steel. JMatPro software was employed to calculate the Time-Temperature-Transformation (TTT) diagram of CSP-50# and TRC-50#-10. The true grain sizes of CSP-50# and TRC-50#-10 specimens were used for TTT diagram calculation. 

## 3. Results

**Figure 2:**
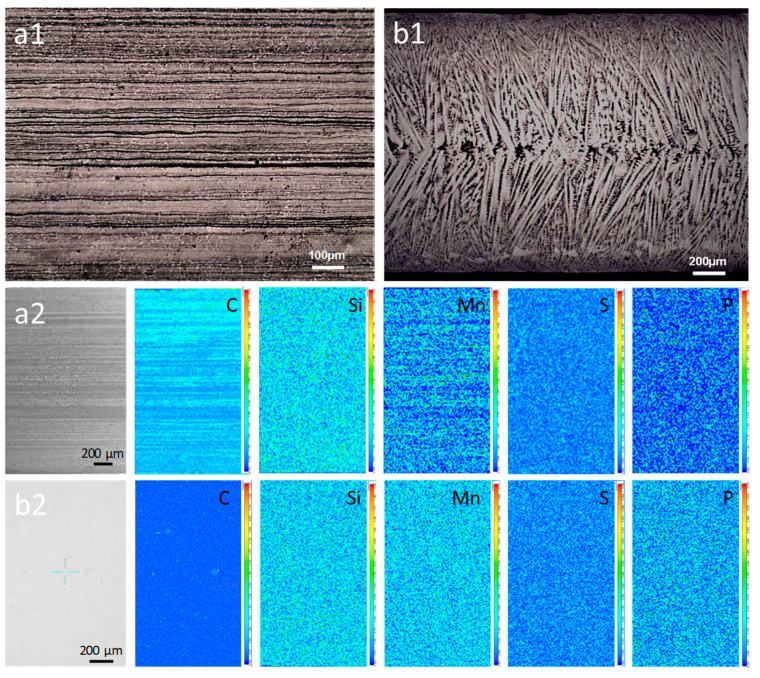
Microstructural analysis and alloying elements mapping of the hot-rolled strips by OM and EPMA. (**a1**,**a2**) CSP-50#. (**b1**,**b2**) TRC-50#-10.

Figure 2(a1) shows the banded segregation and center segregation appeared in the hot-rolled CSP-50#. The dark and bright banded areas in Figure 2(a1) correspond to the alloying element rich and poor regions, respectively. The width of the central composition segregation zone is larger than that of the other positions, indicating that heavier segregation occurred in the central area. EPMA analysis shows that apparent C and Mn segregation happened, while the segregation extent of Si is slight (Figure 2(a2)). The remaining elements (P, and S) are distributed uniformly (Figure 2(a2)). Owing to the characteristic of single-pass hot rolling, the TRC-50#-10 sample shows a typical dendritic microstructure with a fine dendrite arm spacing (Figure 2(b1)). The average primary and secondary dendrite arm spacings (SDAS) are 36.89 μm and 16.94 μm, respectively. The SDAS of TRC is much smaller than those of the steel strips produced by the CSP process (ranging from 32 μm to 120 μm) and the conventional process (ranging from 100 μm to 300 μm) [8]. EMPA analysis of the TRC-50#-10 sample shows that no obvious composition segregation is observed (Figure 2(b2)), and all the alloying elements are uniformly distributed, indicating that no banded segregation or evident dendritic segregation exists in the sample.

**Figure 3:**
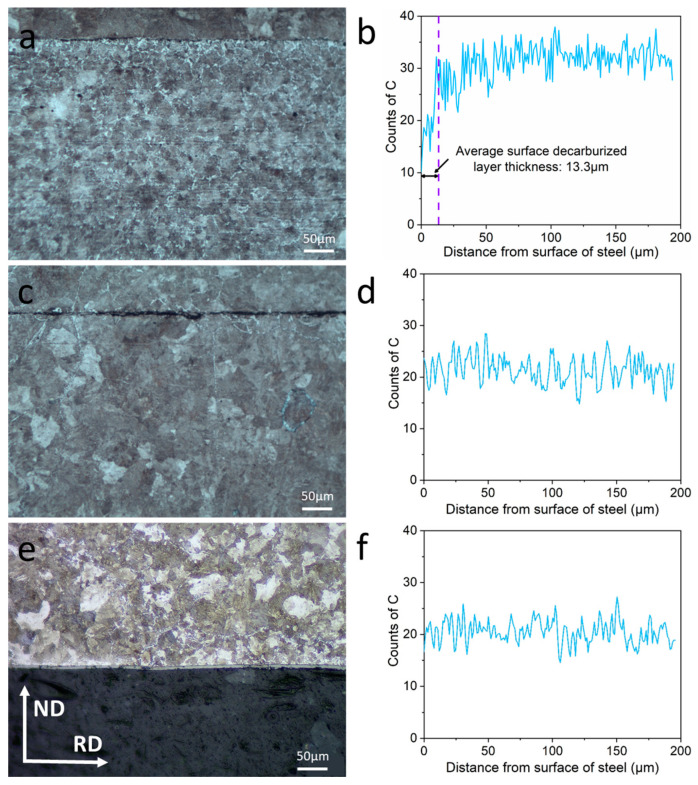
Optical micrographs and EPMA composition profiles of C from surface to inside of the hot-rolled strips. (**a**,**b**) CSP-50#. (**c**,**d**) TRC-50#-10. (**e**,**f**) TRC-50#-33.

The optical micrographs of CSP-50#, TRC-50#-10 and TRC-50#-33 and their typical C concentration profiles from the surface to the interior are shown in Figure 3. The ferrite fraction of the CSP-50# gradually decreases from the surface to the interior, indicating that there is a partial decarburization layer on the sample surface, as shown in Figure 3a. The typical C concentration profile shown in Figure 3b reveals that for the CSP-50#, the concentration of C increases gradually from the surface to the interior. The average surface decarburization layer thickness of the CSP-50# is 13.3 μm. In contrast, the optical micrographs in Figure 3c,e show that the ferrite fraction of TRC-50#-10 and TRC-50#-33 almost remain unchanged from the surface to the interior. The EPMA results in Figure 3d,e show that the C concentration of TRC-50#-10 and TRC-50#-33 remains unchanged with an increase in distance from the surface to the interior. Thus, it indicates that there is no obvious decarburization on the surface of TRC-50#-10 and TRC-50#-33.

**Figure 4:**
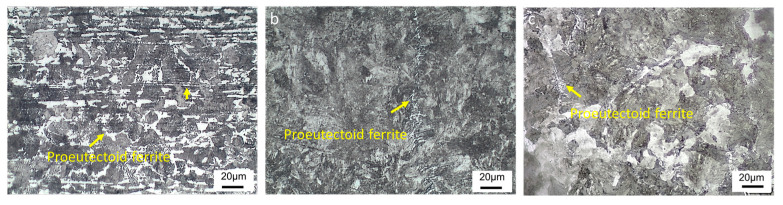
Optical micrographs of the hot-rolled strips. (**a**) CSP-50#. (**b**) TRC-50#-10. (**c**) TRC-50#-33.

Figure 4 illustrates microstructures of CSP-50#, TRC-50#-10 and TRC-50#-33. The microstructure of CSP-50# consists of ferrite networks and ferrite bands (bright) alternating with pearlite bands (black), as shown in Figure 4a. The micrographs in Figure 4b,c show that the microstructures of TRC-50#-10 and TRC-50#-33 consist of pearlite and grain boundary ferrite networks (proeutectoid ferrite). Table 4 presents the microstructural parameters of the hot-rolled strips. The ferrite volume fraction of CSP-50# is 18%, while the ferrite volume fractions of TRC-50#-10 and TRC-50#-33 are lower than 1% and 2%, respectively. In addition, the higher volume fraction of ferrite in TRC-50#-33 indicates that the increase in rolling reduction promotes the ferrite formation. Owing to the characteristic of single-pass hot rolling in the TRC process, the grain refinement of prior austenite grains for TRC-50#-10 strips is limited. The PAGS of CSP-50# is 24 μm, while for TRC-50#-10 and TRC-50#-33, the PAGSs are 164 μm and 70 μm, respectively (Table 4), which suggests that increasing the rolling reduction in the TRC process can significantly refine the PAGS.

**Figure 5:**
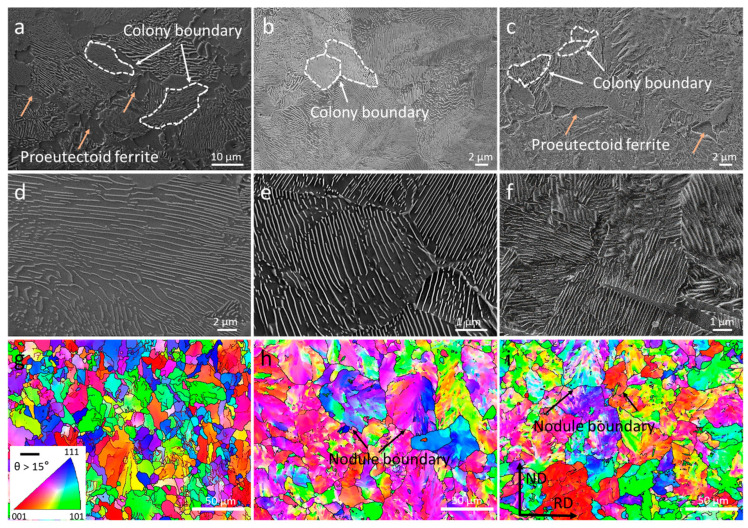
SEM and EBSD micrographs of three hot-rolled strips. (**a**–**c**) SEM images of CSP-50#, TRC-50#-10 and TRC-50#-33 showing the formation of proeutectoid ferrite and pearlite structure. (**d**–**f**) SEM images of CSP-50#, TRC-50#-10 and TRC-50#-33 showing the characteristics of their pearlitic structure. (**g**–**i**) Inverse pole figure (IPF) maps of CSP-50#, TRC-50#-10 and TRC-50#-33 showing the distribution of pearlite nodule. Some of the colony boundaries are marked with white dashed lines. The proeutectoid ferrites are marked by brown arrows. The nodule boundaries are marked by black arrows.

**Figure 6:**
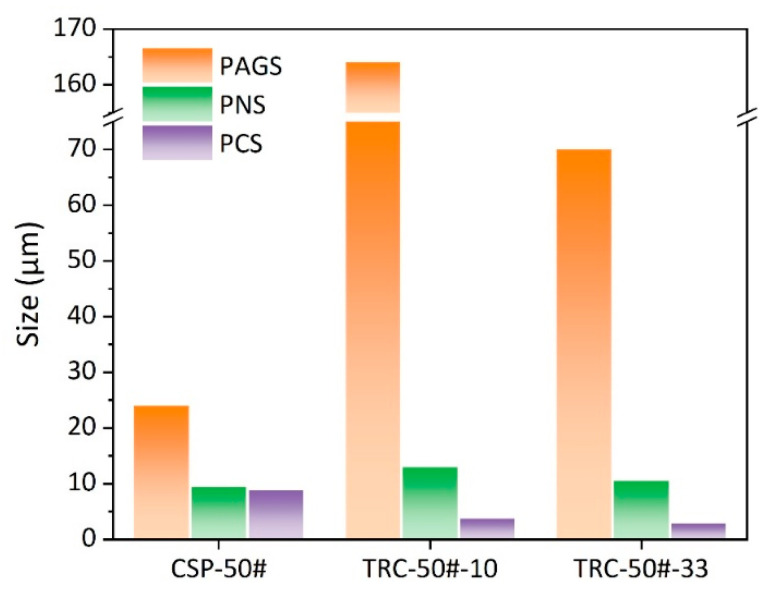
The PAGS, PNS and PCS distributions of the three hot-rolled strips.

SEM and EBSD micrographs in Figure 5 show the microstructure of pearlite (pearlite colony and pearlite nodule) for CSP-50#, TRC-50#-10 and TRC-50#-33. Pearlite colony refers to a region in which cementite lamellas have nearly the same direction (highlighted by white dashed lines), as shown in Figure 5a–c. Compared with the TRC-50#-10 and TRC-50#-33, the CSP-50# has more proeutectoid ferrite distributed around pearlite colony (Figure 5a–c). Pearlite nodules nucleate at austenite grain boundaries (highlighted by black arrows), where ferrite lamellae have nearly the same crystallographic orientation [49]. The nodule size (proeutectoid ferrite can be included in the measurement of the nodule size) is determined using the inverse pole figure (IPF) maps as presented in Figure 5g–i. Table 4 shows the microstructural parameters of the three hot-rolled strips. The PNS of CSP-50# steel is smaller than PCS because the microstructure contains proeutectoid ferrite with a volume fraction of 18% and average grain size of 4.3 μm. CSP-50# exhibits smaller PNS, larger PCS, and IS compared with TRC-50#-10 and TRC-50#-33. As shown in Figure 6, the PCS and PNS of TRC-50#-33 are larger than those of TRC-50#-10, which indicates that the smaller the PAGS, the smaller the PCS and PNS. However, the measured IS is similar for TRC-50#-10 and TRC-50#-33 due to their identical coiling temperature (Table 3).

**Figure 7:**
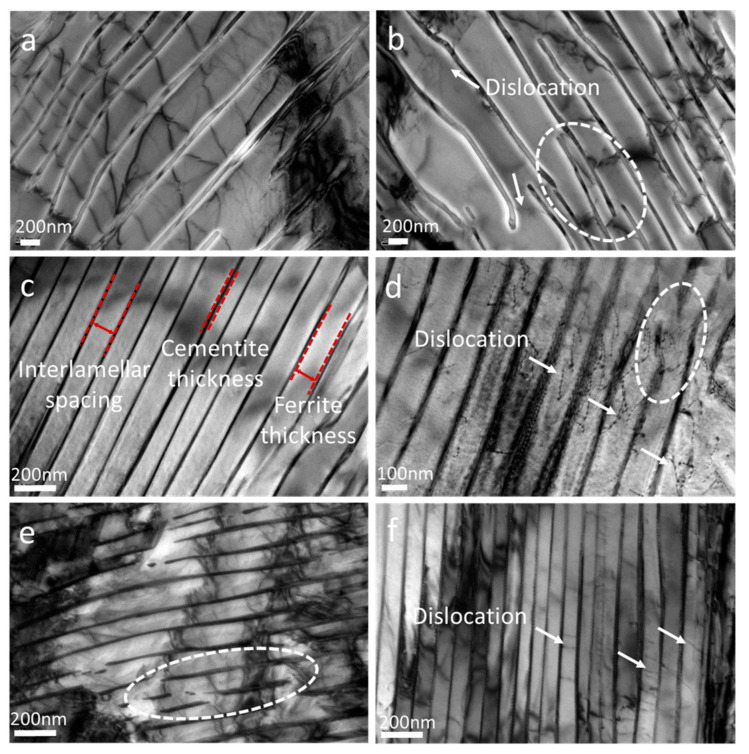
TEM micrographs showing the pearlite structure of the three hot-rolled strips. (**a**,**b**) CSP-50#. (**c**,**d**) TRC-50#-10. (**e**,**f**) TRC-50#-33. White dashed lines and arrows highlight the distribution of fractured cementite and dislocation, respectively.

**Figure 8:**
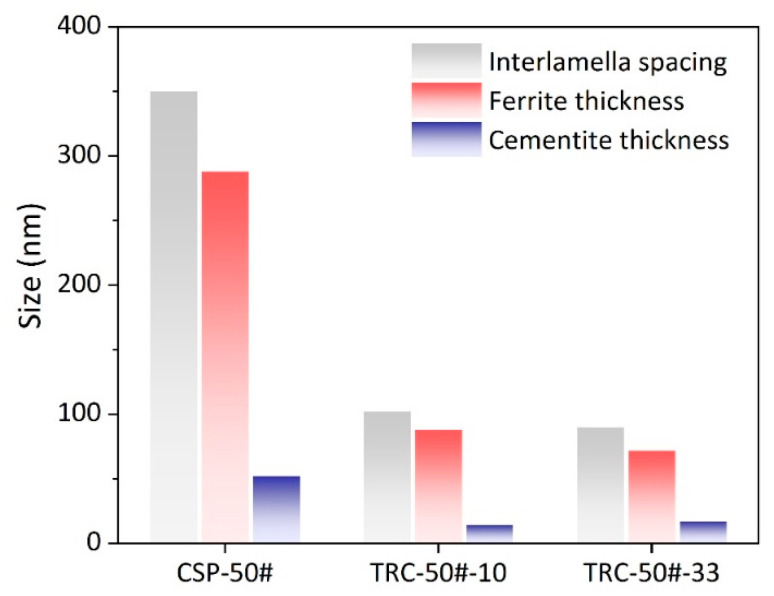
The statistics of the pearlite IS, the thickness of ferrite lamella, and the thickness of cementite lamella of the three hot-rolled strips with edge-on condition.

Figure 7 and Figure 8 present the TEM micrographs of CSP-50#, TRC-50#-10 and TRC-50#-33, and the corresponding statistics of IS, respectively. Compared with the CSP-50#, much finer lamellas are obtained for the TRC-50#-10 and TRC-50#-33 (Figure 7a,c,e). These results are consistent with the SEM results (Figure 5). For the TRC-50#-10 and TRC-50#-33, their ferrite lamella thicknesses are similar, so are their cementite lamella thicknesses. The PFT and PCT are finer than those of CSP-50# (Table 4). As for the three hot-rolled strips, the growth direction of cementite changes (highlight by white dashed lines in Figure 7b,d,e) during the process of growth. This should be related to the presence of dislocations in the ferrite lamellar (highlighted by white arrows) that affect the growth of the cementite lamella [50]. It is noted that the existence of dislocations in the ferrite lamellar could be related to the different thermal expansion coefficients of the ferrite and cementite during the transformation process, which cause different volume expansions of the two phases [49,51]. Statistical results indicate that CSP-50# has the largest IS, followed by TRC-50#-10 and TRC-50#-33, and the thicknesses of ferrite lamella and cementite lamella increase with increasing IS.

## 4. Discussion

### 4.1. Composition Segregation of the Hot-Rolled Strips

There are obvious C-Mn segregation bands in the RD-ND plane of the CSP-50# strip, especially in the center (Figure 2). During continuous casting, solute partition occurs in the mushy zone between solid and liquid phases, leading to the formation of inter-dendritic regions rich in solute elements. The partition coefficient (k) is used to express the tendency of solute being rejected from the solid phase to the liquid phase, which is usually smaller than 1.0. Liu et al. reported that the smaller the partition coefficient, the heavier the segregation degree, whilst for the dendrite, the finer the inter-dendritic space, the smaller the amount of residual liquid remaining in the inter-dendritic regions [8]. Assuming that at a certain temperature, the solute concentrations of the solid and liquid phases are denoted as C_s_ and C_L_, respectively, the solute equilibrium partition coefficient (k) is defined as [52]:

(1) $$k=C_{s}/C_{L},$$

**Figure 9:**
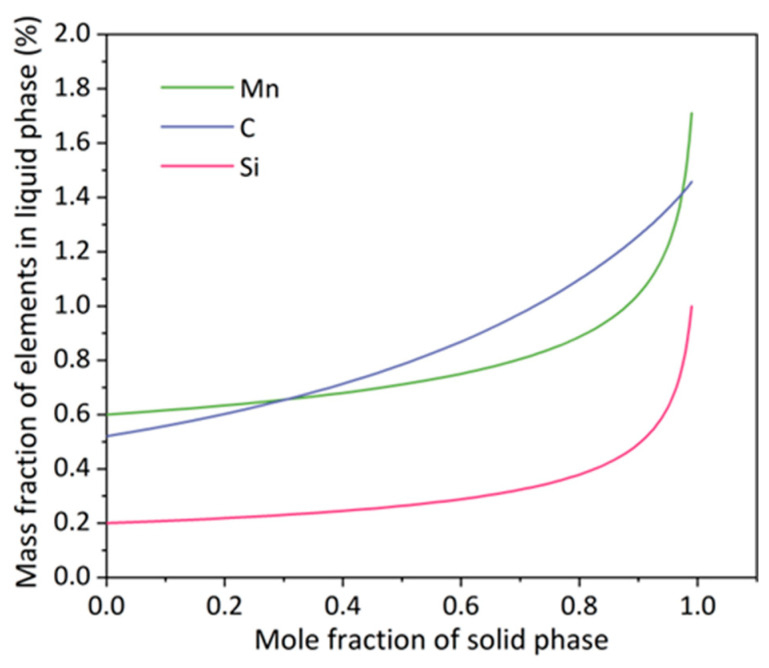
Composition segregation profiles across a dendrite of 50# steel calculated by Thermo-Calc using the Scheil solidification model.

In addition, the solute concentration C_s_ in the solid phase and the solid phase volume fraction, f_s_, obey the Scheil equation, as follows:

(2) $$C_{s}=kC_{0}{\left({1-f}_{s}\right)}^{k-1},$$

where C_0_ is the initial solute concentration of the alloy. The solute equilibrium partition coefficients of C are the smallest, resulting in more C rejected to the residual liquid [8]. This indicates that the segregation degree of the C is more serious than that of Mn and Si, consistent with the composition segregation of CSP-50# in Figure 2(a2). Although the equilibrium partition coefficient of Mn is high, Mn is generally regarded as an element with high segregation tendency. This is because the content of Mn in steel is relatively high, and its diffusion coefficient in the solid phase is very low [53]. Therefore, Mn segregation is difficult to be eliminated and can be observed easily. As shown in Figure 9, the composition segregation during the solidification of 50# steel is calculated by Thermo-Calc with the Scheil solidification model. The solute concentration in the solid phase is the lowest when the solidification just started. As the solid phase mole fraction increases, the mass fractions of C, Si and Mn in the liquid phase increase gradually. The solute concentration in the solid phase increases as the solidification progresses, and the highest solute concentration is achieved when the last part of the liquid solidifies. Figure 9 illustrates that C starts to enrich in the liquid phase at the beginning of solidification, while Mn and Si are not significantly enriched in the liquid phase until the solid phase mole fraction reaches 80%. More C and Mn enriched at the dendrite front with the further growth of dendrites. Hot rolling changes the shapes of the inter-dendritic areas and renders the bands parallel to the rolling direction, producing an alternating distribution of rich and poor C-Mn regions (Figure 2(a1,a2)). The solidification cooling rate has an important influence on the size of the inter-dendritic space. According to the research [8,54], the higher the solidification cooling rate, the smaller the secondary dendrite arm spacing, and the lower the content of solute elements enriched between the dendrites. The relationship between the cooling rate R (K/s) and the SDAS λ_2_ (μm) in the solidification process is as follows [55]:

(3) $$\lambda_{2}=727{\left(60R\right)}^{-0.41},$$

According to the SDAS measured above, the solidification cooling rates of 50# steel prepared by the TRC process and the CSP process are 160 K/s and 1~34 K/s, respectively. The solidification cooling rate of the TRC process is about 5–100 times higher than that of the CSP process. Additionally, owing to the thermal shrinkage and bulging of CSP-50#, the inter-dendritic liquid is driven to flow towards the centerline [8,56]. Moreover, the SDAS in the center of the CSP-50# slab is larger than that on the surface [8]. Therefore, the segregation at the center zone of CSP-50# is much heavier (Figure 2(a1,a2)). Although the dendrite morphology can be observed in Figure 2(b1), no composition segregation was observed in the EPMA results of TRC-50#-10 (Figure 2(b2)), suggesting that the strips produced by the TRC process have more uniform element distribution than CSP-50#. Therefore, the TRC process has great advantages in inhibiting the elements segregation.

### 4.2. Surface Decarburization of the Hot-Rolled Strips

Decarburization occurs when carbon atoms on the surface interact with the heated atmosphere and are removed from the steel as a gaseous phase, resulting in a lower carbon content on the surface. For medium carbon steels, Zhang and Liu et al. reported that only a partial decarburization layer was observed when the soaking temperature is in the range of 950 °C~1200 °C [3,24,25]. Since the soaking temperature for CSP-50# is in the range of 1150 °C~1180 °C (soaking time is 25~35 min) (Figure 1b), this explains the occurrence of a partial decarburization layer in the hot-rolled CSP-50# strip (Figure 3a,b). As the range of soaking temperature is higher than the TG temperature which is the A_3_ temperature for pure iron (C = 0), the formation of a partial decarburization layer occurs at the single-phase region [3,18]. The carbon concentration can change freely in this austenite single-phase region, and the carbon content on the surface is lower than that in the interior of the strip with the increase in soaking temperature and time. The lower carbon content will unavoidably decrease the stability of the undercooled austenite. Therefore, ferrites nucleate firstly at austenite grain boundaries in the surface area during the coiling. In addition, the long hot rolling of the CSP process will also facilitate surface decarburization [24]. For TRC-50#-10 and TRC-50#-33, there is a protective atmosphere between strip casting and hot rolling (Figure 1a), which greatly reduces the interaction between cast strip surface and oxygen, which can substantially inhibit the occurrence of decarburization [3]. Furthermore, owing to the absence of the high temperature soaking process, the fast hot rolling rate of the single-pass hot rolling process [24] and the high cooling rate after rolling [25], decarburization does not occur in the TRC-50# hot-rolled strip.

### 4.3. Phase Transformation of the Hot-Rolled Strips

**Figure 10:**
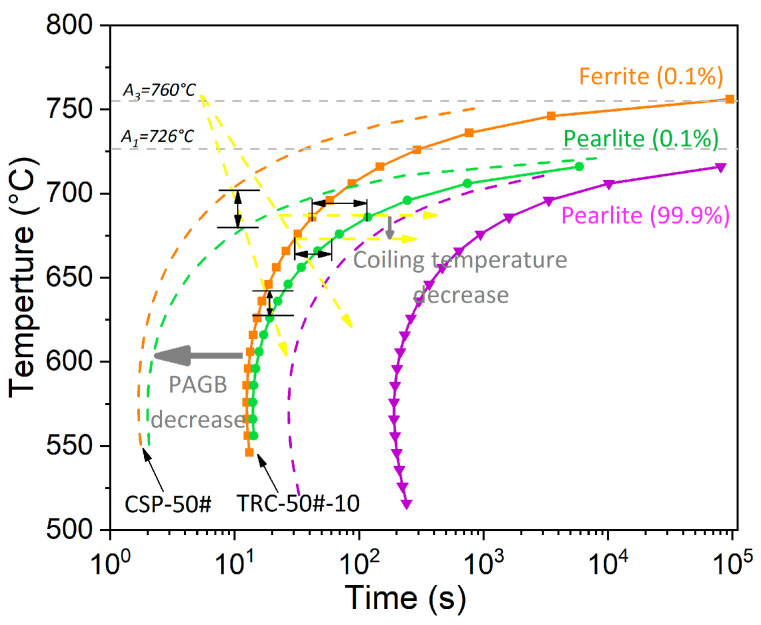
Time-Temperature-Transformation (TTT) diagram of 50# steel calculated by the JMatPro software. Dashed lines and solid lines correspond to CSP-50# (PAGS: 24 μm) and TRC-50#-10 (PAGS: 164 μm), respectively. Brown curves indicate the ferrite transformation start temperature. Green curves indicate the ferrite transformation finish transformation and pearlite transformation start temperature. Purple curves indicate the pearlite transformation finish transformation. Brown, green and purple curves shift to the left after PAGS refinement. Yellow dotted lines with arrows indicate the phase transformation process. Black double-arrow solid lines indicate the width of the two-phase region of ferrite and pearlite during the phase transformation.

CSP-50# shows an obvious band-like pearlite–ferrite structure, while in TRC-50#-10 and TRC-50#-33 the microstructures consist of pearlite and grain boundary ferrite network (Figure 4). Studies [9,10,11] have shown that the reason for the formation of the typical band-like pearlite–ferrite structure is the inter-dendritic micro-segregation. In addition, austenite grain size and cooling conditions are also responsible for the formation of band-like structures [10,57]. After the hot rolling, the segregation of C and Mn distributed along the rolling direction on the RD-ND plane of the strip produced by CSP, forming band-shaped C-Mn enriched and poor regions. In addition, Mn increases the diffusion activation energy of C and thus reduces the diffusion rate of C. Therefore, the Mn-rich region tends to attract C, which slows down the diffusion of C [52], leading to the increase in the stability of supercooled austenite and a decrease in the austenite to ferrite transformation temperature [9,52]. Moreover, it is well known that austenite grain refinement and high phase transformation temperature both favor the formation of banded ferrite and pearlite [57,58]. As the CSP-50# has a fine grain size of 24 μm and a high coiling temperature of 700 °C, ferrite preferentially develops along Mn-depleted regions, while pearlite forms in the high-Mn regions. However, owing to the uniform distribution of alloying elements, no band-like structure was observed for the TRC-55#-10 and TRC-55#-33. Figure 3 and Table 4 show that the volume fraction of pearlite for CSP-50# is lower than those of TRC-50#-10 and TRC-50#-33 because the refined austenite grain size of CSP-50# and the high transformation temperature promote the austenite to ferrite phase transformation as proeutectoid ferrite prefer to nucleate at austenite grain boundaries [58,59]. As displayed in Figure 10, reducing the PAGS and increasing the phase transformation temperature increase the width of the two-phase region of austenite-ferrite, and then more supercooled austenite will transform into ferrite.

The PCS and PNS of TRC-50#-33 are larger than those of TRC-50#-10 (Figure 6), which is attributed to the small PAGS of TRC-50#-33 [35,37,38,60,61]. However, the PNS is almost independent of the coiling temperature [47]. Previous literature show that the PCS decreases with the PAGS [41,42,43,44,45,48]. The refinement of austenite grain increases the sites for pearlite nucleation and, hence, reduces the colony and nodule size. However, although CSP-50# has the smallest PAGS, in comparison with TRC-50#-10 and TRC-50#-33, it has the largest PCS (Figure 6), indicating that coiling temperature also affects the PCS [47]. It has been reported that PCS decreases with isothermal transformation temperature [47,58]. The refinement of the PCS is related to the increase in cementite nucleation sites at greater supercooling conditions. Figure 6 shows that TRC-50#-10 and TRC-50#-33 share similar IS, which is finer than that of CSP-50#. For isothermal transformation, the effect of PAGS on IS is negligible [58]. The degree of supercooling plays a decisive role in IS because the lower the phase transformation temperature and diffusivity are, the smaller the IS will be [47]. The IS and the pearlite transformation temperature satisfies the following equations [62]:

(4) $$\Delta T=\frac{T_{e}{-P}_{s}}{T_{e}},$$

where ΔT, T_e_ and P_s_ represent the degree of undercooling, the equilibrium transformation temperature and the pearlite transformation temperature, respectively.

(5) $$\lambda =\frac{2\sigma }{V_{M}\cdot \Delta H}\cdot \left(\frac{T_{e}}{T_{e}-P_{s}}\right)=\frac{2\sigma }{V_{M}\cdot \Delta H}\cdot \frac{1}{\Delta T}=a\cdot \frac{1}{\Delta T},$$

where λ is IS; ΔH is the enthalpy change in the pearlite transformation; V_M_ is the molar volume of pearlite; σ is the interfacial energy between ferrite and cementite; a is a constant.

## 5. Conclusions

In this paper, through the quantitative and comparative analysis of the segregation, decarburization and phase transformation of medium carbon steel strips produced by the TRC and CSP processes, the effects of the TRC and CSP processing parameters on the segregation, decarburization and phase transformation of 50# steel were investigated, and the conclusions are as follows:

(1) The TRC process has great advantages in controlling elements segregation for medium carbon steel strip production. CSP-50# shows distinct band segregation of C and Mn, especially the central segregation, but no composition segregation was observed in TRC-50#-10 and TRC-50#-33 strips;
(2) No apparent decarburization was observed on the surface of 50# steel strip produced by the TRC process, while incomplete decarburization with an average thickness of 13.52 μm was observed on the surface of 50# steel strips produced by the CSP, which is partially attributed to the high soaking temperature and long soaking time of the CSP process;
(3) The band segregation of C and Mn of CSP-50# strip induces band-like pearlite–ferrite structure. The steel strip produced by TRC has a higher pearlite volume fraction, larger PNS and smaller PCS and IS due to the co-influence of larger PAGS and the lower coiling temperature.

## Figures and Tables

**Table 1:** Comparison of technical characteristics of the TRC and CSP processes [30].

Process	CSP	TRC
Production line length/m	180–400	~50
Casting speed/(m/min)	3.5–7.0	60–120
Casting slab thickness/mm	50–130	1.4–2.1
Cooling rate/(°C/s)	10^1^–10^2^	10^2^–10^3^
Rolling pass	≥5	1

**Table 2:** Chemical compositions of the investigated CSP-50#, TRC-50#-10 and TRC-50#-33 strips (wt.%).

Sample Code	C	Si	Mn	P	S
CSP-50#	0.51	0.27	0.63	≤0.012	≤0.003
TRC-50#-10	0.49	0.22	0.63	<0.02	<0.003
TRC-50#-33					

**Table 3:** Thermo-mechanical processing parameters of the experiment strips.

Sample Code	Total Rolling Reduction	Coiling Temperature
CSP-50#	98%	700 °C
TRC-50#-10	10%	620 °C
TRC-50#-33	33%	620 °C

**Table 4:** Microstructural parameters of the three hot-rolled strips.

Sample	VFPF/%	PAGS/μm	PNS/μm	PCS/μm	IS/nm
CSP-50#	18	24	7.7 ^1^	8.9 ± 4	350 ± 134
TRC-50#-10	0.8	164	11.6	3.8 ± 2	98 ± 20
TRC-50#-33	2	70	8.6	2.9 ± 1	90 ± 23

^1^ Proeutectoid ferrite was included in the measurement of the nodule size.

## Data Availability

Not applicable.
